# Supplementary material for: Impact of Selected Meteorological Factors on COVID-19 Incidence in Southern Finland during 2020–2021
Source: Int J Environ Res Public Health. 2022 Oct 17;19(20):13398. doi: 10.3390/ijerph192013398 (PMC9603127; doi:10.3390/ijerph192013398)
Supplement: Supplementary file 1 [file ijerph-19-13398-s001.zip › ijerph-1905992-supplementary.pdf]

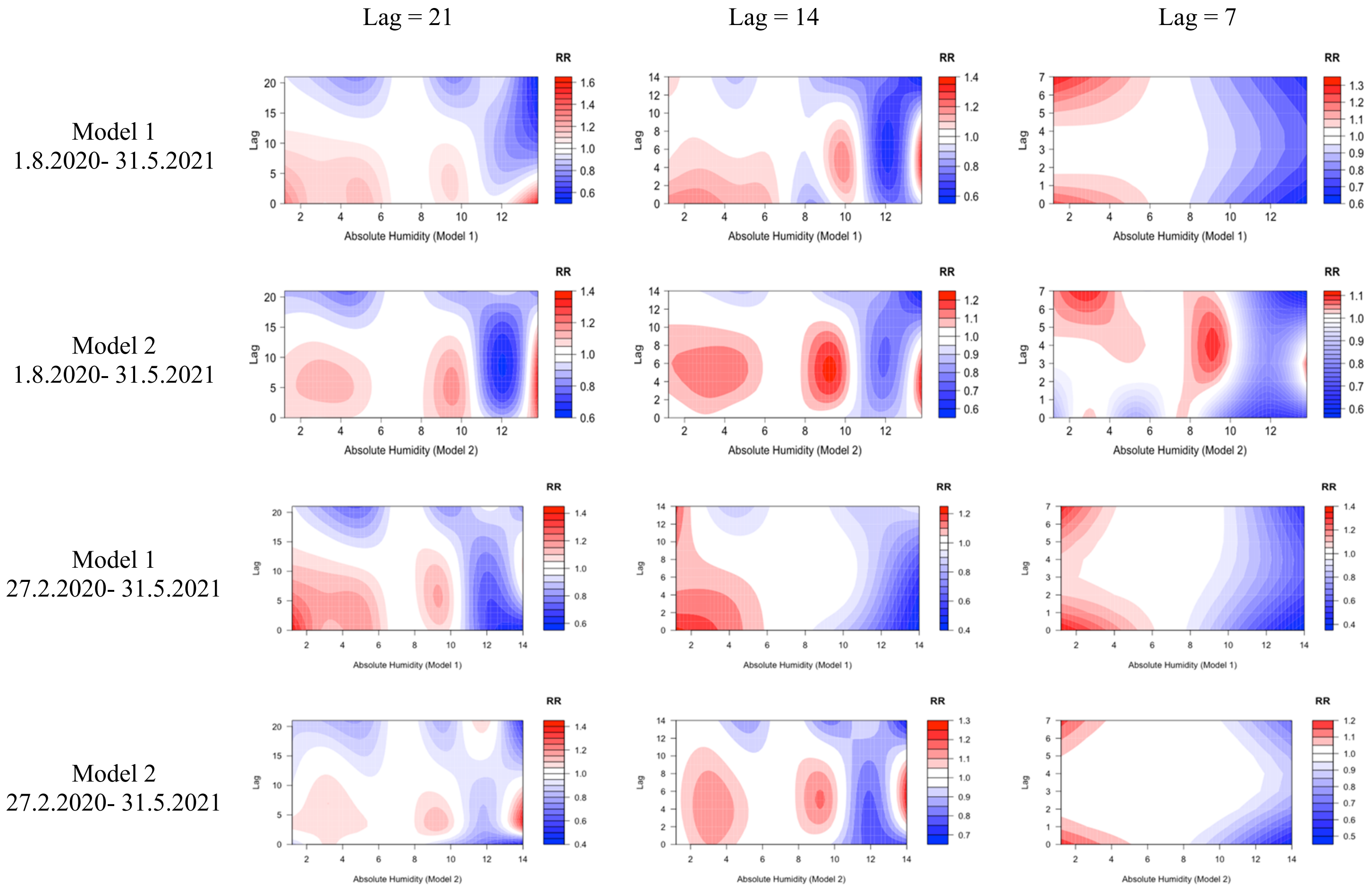
Lag = 21
Lag = 14
Lag = 7
Model 1
1.8.2020- 31.5.2021
Model 2
1.8.2020- 31.5.2021
Model 1
27.2.2020- 31.5.2021
Model 2
27.2.2020- 31.5.2021
RR
Lag
Absolute Humidity (Model 1)
Absolute Humidity (Model 2)

Figure S1. Relative risks (RR) of COVID-19 by daily average absolute humidity and lag, compared to the reference value at 7 g/m³. The figures present RR for Model 1 and Model 2 with 21-,14- and 7 days lag at the Helsinki-Uusimaa hospital district (HUS) from 1 August 2020 until 31 May 2021 or from 27 February 2020 until 31 May 2021. The number of knots at the exposure-response function were based on the lowest AIC with a maximum limit of 6 knots.

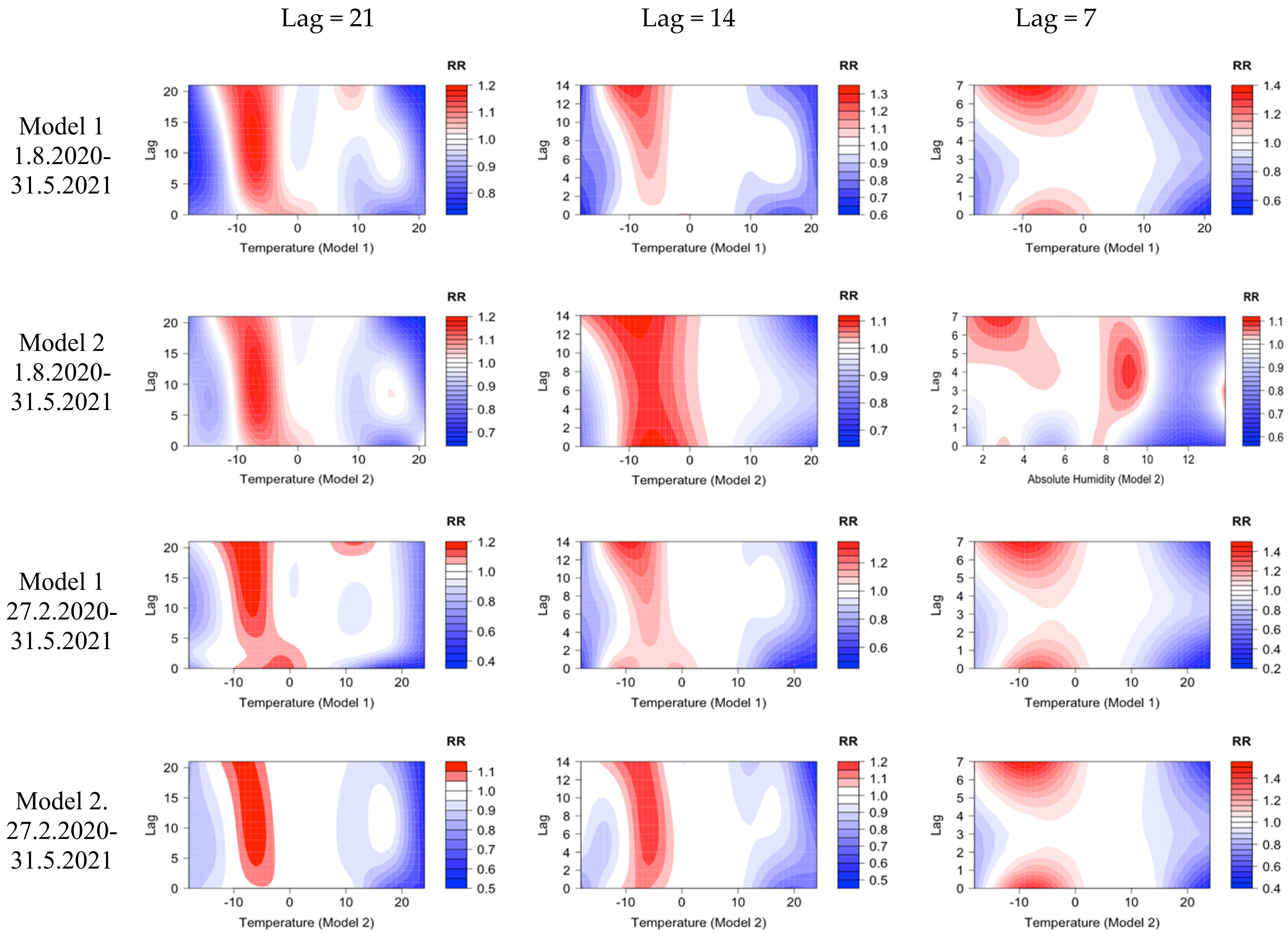

Figure S2. Relative risks (RR) of COVID-19 by daily mean temperature and lag, compared to the reference value at 5°C. The figures present RR for Model 1 and Model 2 with 21-,14- and 7 days lag at the Helsinki-Uusimaa hospital district (HUS) from 1 August 2020 until 31 May 2021 or from 27 February 2020 until 31 May 2021. The number of knots at the exposure-response function were based on the lowest AIC with a maximum limit of 6 knots.

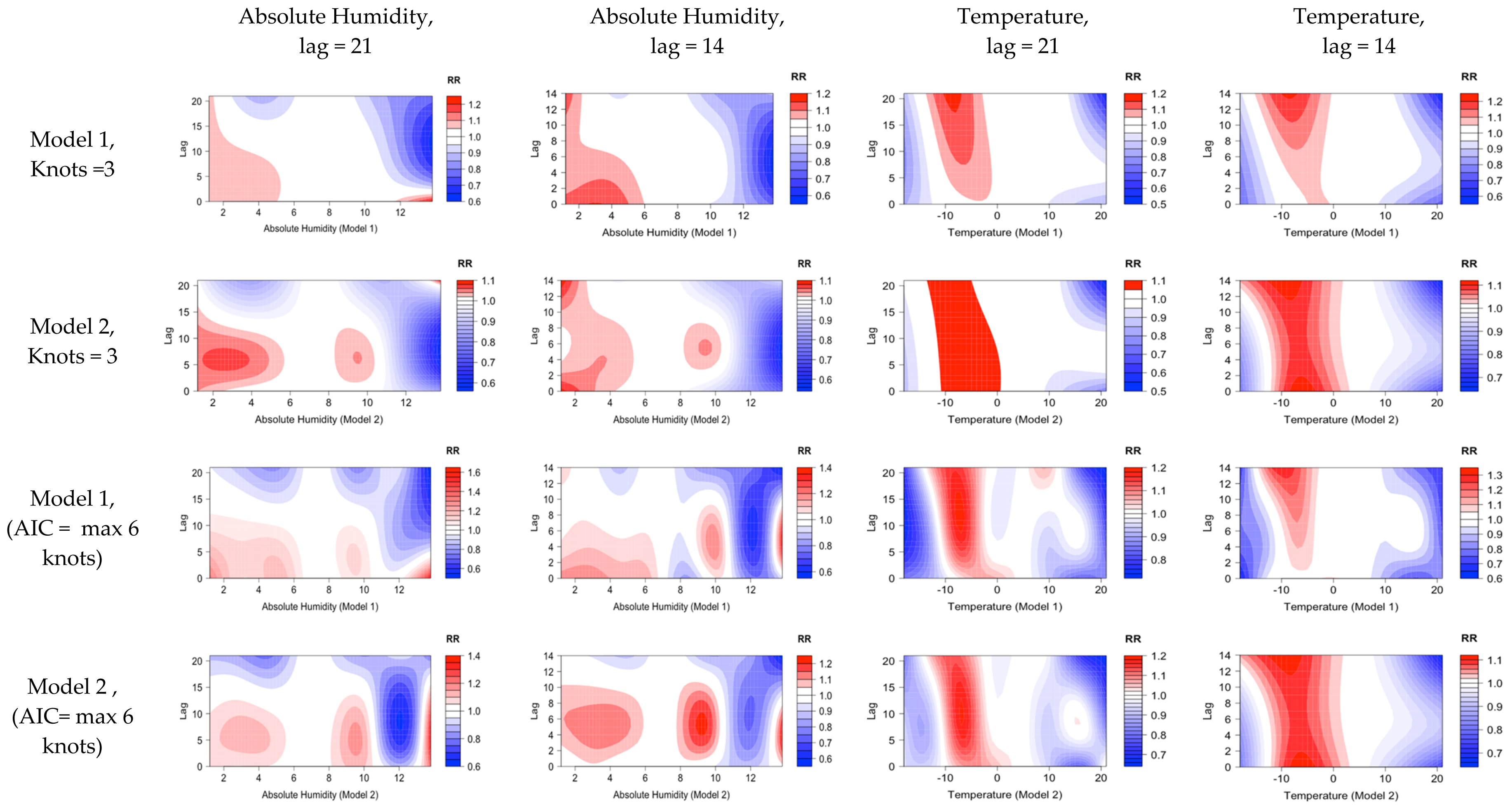

Figure S3. Relative risks (RR) of COVID-19 by daily absolute humidity, mean temperature, and lag, compared to the reference values at 7 g/m³ and at 5°C. The figures present RR for Model 1 and Model 2 with 21- and 14-days lag at the Helsinki-Uusimaa hospital district (HUS) from 1 August 2020 until 31 May 2021. The number of knots for the exposure-response function were fixed at either 3 knots or based on the lowest AIC with a maximum limit of 6 knots.

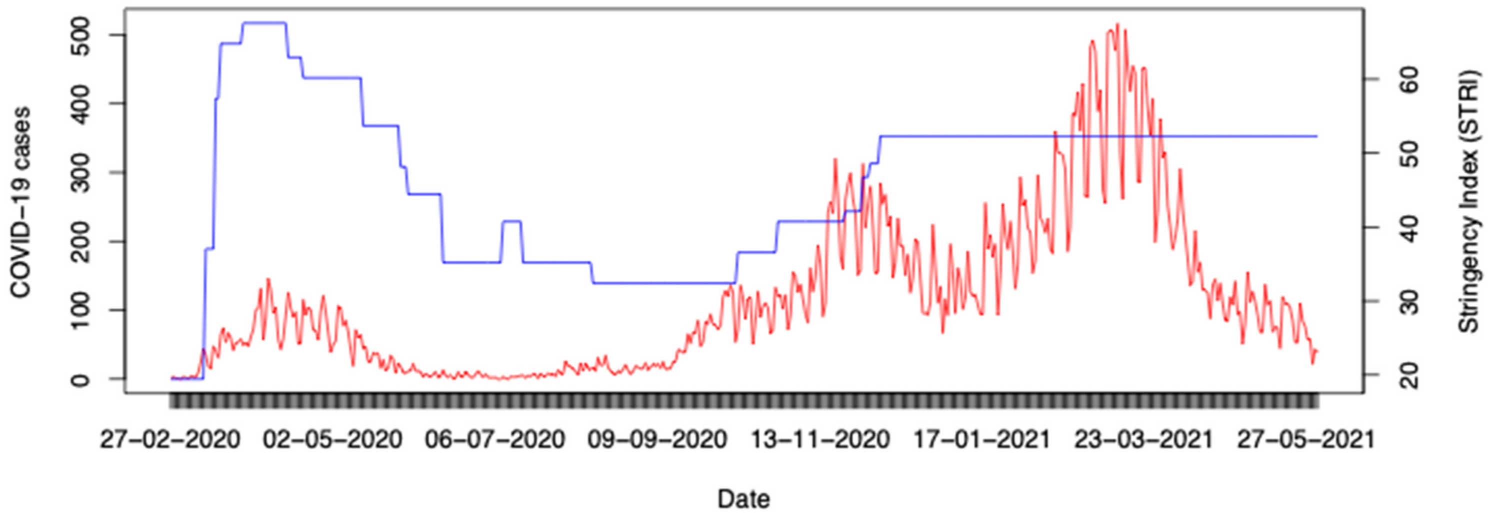

Figure S4. The Stringency Index (STRI) for Finland and daily confirmed cases of COVID-19 for the Hospital District of Helsinki and Uusimaa (HUS) from 27 February 2020 to 31 May 2021. The stringency index is a composite measure that is based on nine response indicators including travel bans, school closures and workplace closures, rescaled to a value from 0 to 100 (100=strictest).
